# Analysis of historical road accident data supporting autonomous vehicle control strategies

**DOI:** 10.7717/peerj-cs.399

**Published:** 2021-02-23

**Authors:** Sándor Szénási

**Affiliations:** 1 Faculty of Economics and Informatics, J. Selye University, Komárno, Slovakia; 2 John von Neumann Faculty of Informatics, Óbuda University, Budapest, Hungary

**Keywords:** Data mining, DBSCAN, Road accident, Statistics, Autonomous vehicle, Road safety

## Abstract

It is expected that most accidents occurring due to human mistakes will be eliminated by autonomous vehicles. Their control is based on real-time data obtained from the various sensors, processed by sophisticated algorithms and the operation of actuators. However, it is worth noting that this process flow cannot handle unexpected accident situations like a child running out in front of the vehicle or an unexpectedly slippery road surface. A comprehensive analysis of historical accident data can help to forecast these situations. For example, it is possible to localize areas of the public road network, where the number of accidents related to careless pedestrians or bad road surface conditions is significantly higher than expected. This information can help the control of the autonomous vehicle to prepare for dangerous situations long before the real-time sensors provide any related information. This manuscript presents a data-mining method working on the already existing road accident database records to find the black spots of the road network. As a next step, a further statistical approach is used to find the significant risk factors of these zones, which result can be built into the controlling strategy of self-driven cars to prepare them for these situations to decrease the probability of the potential further incidents. The evaluation part of this paper shows that the robustness of the proposed method is similar to the already existing black spot searching algorithms. However, it provides additional information about the main accident patterns.

## Introduction

Human drivers have many disadvantages compared to autonomous vehicles (slower reaction time, inattentiveness, variable physical condition) (Kertesz & Felde, 2020). Nevertheless, they can often perform better (Chatterjee et al., 2002) in some unexpected situations like a child running out in front of the vehicle. Because beyond the information gained in real-time, they may have specific knowledge about a given location (linked to the previous example, the human driver may know that there is a playground without a fence near the road; therefore, the appearance of a child is not unexpected). Drivers also have some incomplete but useful historical knowledge about accidents and they can build this information into their driving behavior. If they know that there were several pedestrian collisions somewhere, they will decrease the speed and try to be more attentive without triggering real-time signals. Thanks to this behavior, they can prepare for and avoid some types of accidents, which were not possible without this historical data. Another example might be a road section, which is usually extremely slippery on rainy days. Real-time sensors can detect the element of slipping when it is too late to avoid the consequences. Some historical accident data can help to prepare the car for these unexpected situations.

We propose the following consecutive steps to integrate historical data into the control algorithm for autonomous devices:

1. Localize accident black spots in an already existing accident database, using statistical or data-mining methods;
2. Determine the common reasons for these accidents with statistical analysis or pattern matching;
3. Specify the necessary preventive steps to decrease the probability of further accidents.

This article mainly focuses on the first two steps because the third one largely depends on the limits and equipment of the self-driven car. For example, in the case of dangerous areas is it possible to increase the power of lights to make the car more visible? Or in the case of large chance of pedestrian accidents, is it possible to increase the volume of the artificial engine sound to avoid careless road crossing? Can the car change the suspension settings to prepare for potentially dangerous road sections? The scope of this paper is the development of the theoretical background to support these preliminary protection activities.

The appropriate preliminary actions may significantly decrease the number and severity of road accidents. For example, Carsten & Tate (2005) present a model for the relationship between changes in vehicle speed and the number of occurred accidents. It is visible from this model (based on the national injury database of Great Britain to predict the effects of speed on road accidents) that for each 1 km/h change in mean speed, the best-estimated change of accident risk is 3%. Accordingly, it is worth making assumptions about the dangerous areas and adapting the control of the autonomous cars to these predictions.

## Background

### Black spot identification

Black spot management (identification, analysis, and treatment of black spots in the public road network) is one of the most important tasks of road safety engineers. The identification of these extremely hazardous sections is the first step to prevent further accidents or to decrease the seriousness of these. It is a heavily researched area, and there are several theoretical methods for this purpose.

However it has a long tradition in traffic engineering; interestingly, there is not any generally accepted definition of road accident black spots (also known as hot spots), the official definition varies by country. It follows that the method used to find these hazardous locations also varies by country. For example, by the definition of the Hungarian government, outside built-up area black spots are defined as road sections no longer than 100 meters where the number of accidents during the last three years is at least 3. According to this, road safety engineers use simple threshold-based methods (for example, the traditional sliding window technique) to find these areas. Switzerland uses a significantly different definition as black spots are sections of the road network (or intersections) where the number of accidents is “well above” the number of accidents at comparable sites. The key difference is the term “comparable sites” because these advanced comparative methods do not try to classify all road segments by itself but try to compare to similar areas.

There are some general attributes of accident black spots to overcome the conceptual confusion. These are usually well-defined sections or intersections of the public road network, where road accidents are historically concentrated (Elvik, 2008; Delorme & Lassarre, 2014; Murray, White & Ison, 2012; Montella et al., 2013; Hegyi, Borsos & Koren, 2017). Nowadays, road accidents are monitored by the governments and all data about accidents are stored in large, reliable and partially public databases (without any personal information about the participants). Much data about the road network is also available (road layout, speed limits, tables, etc.). As a result, road safety engineers can use several procedures from various fields (statistics, data mining, pattern recognition) to localize accident black spots in these databases.

It is a common assumption that the number of accidents is significantly higher at these locations compared to other sections of the road network. However, this alone is neither a necessary nor sufficient condition. The variation of the average yearly accident count of road sections is relatively high compared to the number of accidents. Because of this, the regression to the mean effect can distort the historical data. A given section with more accidents than average is not necessarily an accident black spot. The converse is also true, as there may be true black spots with relatively few accidents for a given year. However, this deficiency is already theoretically proven as most black spot identification methods are based on the accident numbers of the last few years, simply because this is the best place to start a detailed analysis.

Nevertheless, it is always worth keeping in mind that these locations are just black spot candidates, but it needs further examination to make the right decision concerning them. The best way to do this is via a detailed scene investigation, but it is very expensive and time-consuming. Another theoretical approach can be the analysis of accident data to find some irregular patterns and identify one or more risk factors causing these accidents. Without these, it is possible that the higher frequency of accidents is purely coincidental at a given location and time.

To localize potential accident black spots, the most traditional procedure is the sliding window method (Lee & Lee, 2013; Elvik, 2008; Geurts et al., 2006). The input parameters of the process are the section length and a threshold value. The method is based on the following:

1. Divide the selected road into small uniform sized sections;
2. Count the number of accidents that have occurred in the last few years for each section;
3. Flag the segments where this number is higher than a given threshold as potential black spots.

There are many variants of the proposed traditional sliding window method (Anderson, 2009; Szénási & Jankó, 2007). A potential alternative is to use variable window length. One of its advantages is that it is unnecessary to set the appropriate parameter, but sufficient to give a minimal and maximal value. The method can try several window lengths to find the largest black spots possible. Due to this modification, it can find small local black spots and larger ones too. The traditional sliding window method uses non-overlapping segments, but it is also possible to slide the window with smaller steps than the window size. This leads to a more sensitive method, which can find more black spot candidates. However, it is also necessary to manage the overlapping black spots (considering these as one big cluster, or multiple distinct ones). It is worth mentioning that the method has some additional advantages: it has very low computational demand (compared to the alternatives) and is based only on the road accident database.

The sliding window method is one of the first widely used procedures; therefore, it is based on the traditional road number + section number positioning system (for example, the accident location is Road 66, 12+450 kilometer+meter). This traditional positioning system was the only real alternative in the past. However, in the last decades, the spreading of GPS technology makes it possible to collect spatial coordinates of accidents. This step has several benefits (faster and more accurate localization) but also requires the rethinking of the already existing methods. It is possible to extend the sliding window method to a two-dimensional procedure, but it is not widely used. It is better to seek out better and more applicable methods fitting to the spatial systems given by the GPS coordinates.

From this field, Kernel Density Estimation (KDE) methods are one of the most popular spatial data analysis techniques (Bíl, Andrášik & Janoška, 2013; Flahaut et al., 2003; Anderson, 2009; Yu et al., 2014; Toran & Moridpour, 2015). These have been employed in many research projects to analyze road accidents. KDE methods have the advantages of simple implementation and easy understanding. These also have the benefit to naturally handle the noise of the data (caused by inaccuracy of GPS devices). In general, KDE is used as an estimation of the Probability Random Function of a random variable. From the safety experts’ point of view, the result of the KDE method is the accident density estimation at a given reference point. The procedure has several parameters, like the search radius distance from the reference point (bandwidth or kernel size) and the kernel function.

Several researchers recommend the use of empirical Bayesian methods combining the benefits of the predicted and historical accident frequencies. These models usually analyze the distributions of the already existing historical data from several aspects, and give predictions about the expected accident state. In the Empirical Bayesian method, the existing historical accident count and the expected accident count predicted by the model are added using different weights (Ghadi & Török, 2019). Because of this, this process requires an accurate accident prediction model.

Another group of already available methods is based on clustering techniques. These procedures are from the field of data mining, where clustering is one of the widely used unsupervised learning methods. In this context, a cluster is a group of items, which are similar to each other and differ from items outside the cluster. Accidents with similar attributes (where properties can be the location and/or another risk factor(s)) can be considered as one cluster, using this concept in the field of black spot searching. Most studies use the basic K-means clustering method (Mauro, De Luca & Dell’Acqua, 2013), but there are also some fuzzy-based C-means solutions.

As already mentioned, the results of the proposed methods are just a set of black spot candidates. It needs further analysis to make a final, valid decision as to whether it is a real accident black spot or not. Furthermore, whether or not it requires any actions. This is the point where our research turns away from traditional road safety management work (identification and elimination of black spots). Based on the collected clusters, road safety engineers must select the black spot candidates having the largest safety potential, which is based on the prediction of the effect of the best available preventive action (cost of the local improvement activity compared to the expected befits in the number and severity of further accidents). From the perspective of autonomous car control, the role of this safety potential is essential. The self-driven car has no options to solve road safety problems. The only important information is the existence of accident black spots and the potential safety mechanisms, which may help to avoid further crashes. As a second difference, from the road safety engineers’ point of view, it is not necessary that the accidents of a given black spot have common characteristics. The hot spot definition of this paper assumes that accidents of a given cluster have similar attributes because this pattern will be the basis of the preventive actions.

The localization of accident black spot candidates is a heavily researched area and there are several fully-automated methods to find these. Nevertheless, the further automatic pattern analysis of these is not as well developed. This phase usually needs a great deal of manual work by human road safety experts (they must travel to the scene and investigate the environment to support their decisions about recommended actions). However, this process is supportable by some general rules but is mostly done manually using the pattern matching capability of the human mind. To fully automate it, it is necessary to make this method applicable to self-driven cars.

According to this objective, this paper focuses on the help for autonomous vehicles to take the appropriate preventive actions to avoid accidents:

- Localize black spot candidates using historical accident database;
- Make assumptions about the common risk factors and patterns of these accidents;
- According to these preliminary results, the autonomous device will know where the dangerous areas are and what preventive actions to take.

### Automated accident prevention

Autonomous vehicles will have several ways to avoid accidents and, therefore, is a hot, widely researched topic. Nevertheless, most papers deal with options existing only in the far future when autonomous devices will be a part of a densely connected network without any human interferences. Real-world implementations are far from this point, but some technologies already exist, although they are not closely related to autonomous vehicles. Currently, implemented accident prevention systems are built into traditional cars as braking assistants, etc. However, it is worth considering these because such methods will be the predecessor of the future techniques applicable to self-driven vehicles.

The two main classes of accident prevention systems are passive and active methods. Passive systems send notifications to the driver about their warnings but do not perform any active operations. On the contrary, active methods have the right to perform interventions (braking, steering, etc.) to avoid accidents. It seems obvious that these prevention systems have a large positive impact on accident prevention, and it has already been proven by Jermakian (2011) that passive methods have significant benefits. There are more than one million vehicle crashes prevented in the USA each year. As Harpen proved (Harper, Hendrickson & Samaras, 2016), the cost-benefit ratio of these systems is also positive.

Brake assist systems are one of the most researched active systems, where the potential benefits are the lower risk of injury, and the less serious injuries of the pedestrians (Rosén et al., 2010). Current forward-looking crash avoidance systems are usually continuously scanning the space in front of the vehicle using various devices (camera, radar, LIDAR, etc.). If any of these detects an unexpected vehicle or pedestrian, the brake assistant system takes the appropriate (preliminary) actions, which can be the enforcement of the braking system or direct autonomous emergency braking. Bálint, Fagerlind & Kullgren (2013) presented very promising results with a test-based methodology for the assessment of braking and pre-crash warning systems. These typically are only using the real-time information given by the vehicle sensors without any knowledge extracted from historical accident data.

Run-time crash prediction models are also related to the topic of this paper. Hossain et al. (2019) presented a comprehensive comparison and review of existing real-time crash prediction models. The basic assumption of these systems is that the probability of a crash situation within a short time window is predictable by the current environmental parameters measured by the sensors. Therefore, most of the already existing methods use only the acquired sensor data to make real-time decisions about potential crash situations. According to this assumption, authors do not use the already existing accident databases as an input to fine-tune the system’s predictions.

The work of Lenard, Badea-Romero & Danton (2014) is closer to the research presented in this paper. They analyzed the common accident scenarios to support the development of autonomous emergency braking protocols. Based on the hierarchical ascending method in two British accident databases filtered by some previously defined conditions (they use only the urban pedestrian accidents that occurred in daylight and with fine weather conditions), attributes of the most common accident scenarios were presented. This paper defines the major accident scenarios and classifies all existing pedestrian accidents into one of these categories. The results of this research would be useful in the training phase of a self-driven vehicle to introduce all possible scenarios to the algorithm.

The objective of Nitsche et al. (2017) is similar, which proposes a novel data analysis method to detect pre-crash situations at various (T- and four-legged) intersections. The purpose of this work is also to support the safety tests of autonomous devices. They clustered accident data into several distinct partitions with the well-known k-medoids procedure. Based on these clusters, an association rules algorithm was applied to each cluster to specify the driving scenarios. The input was a crash database from the UK (containing one thousand junction crashes). The result of the paper contains thirteen crash clusters, describing the main pre-accident situations.

## Materials and Methods

### Black spot candidate localization

#### Density-based spatial clustering of applications with noise

For the black spot candidate localization step, the Density-Based Spatial Clustering of Applications with Noise (DBSCAN) algorithm was used. It is not widely used in the field of road safety engineering; however, it is one of the most efficient density-based clustering methods from the field of data mining. The main objective of density-based clustering tasks is the following: the density of elements within a cluster must be significantly higher than between separate clusters. This principle distinguishes the two distinct classes of elements: items inside a cluster and the outliers (elements outside of any cluster).

According to the road safety task, elements are the accidents in the public road network. These are identified by spatial GPS coordinates and have several additional attributes (time, accident nature, etc.). The general DBSCAN method needs a definition for distance calculation between two elements. In the case of road accidents, the Euclidean distance between the two GPS coordinates was used (black spots are usually spread over a small area. Therefore, it is a good estimation of the real road network distances).

The DBSCAN method requires two additional parameters:

- ε: a radius type variable (meters);
- *MinPts*: the lower limit for the number of accidents in a cluster (accidents).

The main definitions of the DBSCAN algorithm are as follows:

- ε* environment* of a given *x* element is the space within the ε radius of the *x* element;
- *x* is an *internal element* if the ε* environment* of the given *x* contains at least *MinPts* elements (including *x*);
- *x* is *directly densely reachable* from *y* means that *x* is in the ε* environment* of *y* which is an *internal element*;
- *x* is *densely reachable* from *y* if it is accessible through a chain of *directly densely reachable* elements from *y*;
- all points not *densely reachable* from an internal element are the *outliers*;
- if *x* is an *internal element* then it forms a cluster together with all *densely reachable elements* from *x*.

The objective of the process is to find clusters of accidents in the public road network in which all elements are densely connected, and no further expansion is possible. The steps to achieve this are as follows:

1. Select one *internal element* from the accident database as the starting point. This will be the first point of the cluster.
2. Extend the cluster with all *directly densely reachable* elements from any point of the cluster recursively.
3. If it is not possible to extend the cluster with additional points, the cluster can be considered as final (it contains all the *densely reachable* items from the starting point). If this cluster meets the prerequisites for a black spot candidate, it is stored in the result set.
4. Repeat steps 1–3 for all *internal elements* of the database.

The result of the presented procedure is a set of black spot candidates.

The prerequisites of Step 4 can be one or more of the following:

- The number of accidents should be more than a given threshold.
- The accident density of the given area should be more than a given threshold.

The proposed method has several advantages over the traditional methods. Unlike the sliding window algorithm, which analyzes only the accidents of a given road section, the DBSCAN is a spatial algorithm managing all accidents of the database together. This difference would be substantial in the case of junctions where the accidents of the same junction were assigned to different road numbers. This can be especially critical in the case of built-up areas and traffic roundabouts, where the number of connected roads is high.

#### Determination of accident density

One of the benefits of the traditional sliding window method is that it is easy to interpret for human experts. The number of accidents in a given road section is a very informative number. It is also easy to calculate some derived values, like the accident density, which is the number of accidents divided by the length of the road section. This divisor is often extended with the traffic rate or the time period length values. In the case of spatial black spot localization techniques, the definition of road accident density is more complex. These methods are not based on road sections, so division by the section length is not applicable. It is necessary to calculate the area of the black spot to use it as a divisor somehow.

This article proposes a novel method to calculate the area of the region spanned by the black spot accidents. It finds the smallest boundary convex polygon containing all accidents of a given cluster. The density of the black spot will be the number of accidents divided by the area of this polygon. The area is calculated by the Gauss’ area formula Eq. (1).

(1) $$\eqalign{ & {\rm \alpha} (C) = \displaystyle{{\left| {\sum\nolimits_{i = 1}^{n - 1} {x_i}{y_{i + 1}} + {x_n}{y_1} - \sum\nolimits_{i = 1}^{n - 1} {x_{i + 1}}{y_i} - {x_1}{y_n}} \right|} \over 2}  \cr &= \displaystyle{{\left| {{x_1}{y_2} + {x_2}{y_3} + \ldots + {x_{n - 1}}{y_n} + {x_n}{y_1} - {x_2}{y_1} - {x_3}{y_2} - \ldots - {x_n}{y_{n - 1}} - {x_1}{y_n}} \right|} \over 2}}$$

where

- α(*C*): the area of the *C* polygon (cluster);
- *n*: the number of vertices of the polygon;
- (*x*_i_, *y*_i_): the two-dimensional coordinates of the *i*-th vertex of the *C* polygon (where *i* ∈ {1, 2, …, *n*}).

If the number of accidents is less than three, the proposed area concept is not applicable. However, clusters with one or two accidents are usually not considered as black spot candidates. Therefore, this is not a real limitation. In the case of clusters with more than two accidents, the accident rate is calculated as Eq. (2).

(2) $${\rm \rho} (C) = \displaystyle{{|C|} \over {{\rm \alpha} (C)}}$$

where

- ρ(*C*): the accident density of the *C* cluster;
- |*C*|: the number of accidents in the *C* cluster.

The formula requires the sequence of corner coordinates of the polygon in a given order (in this case, a clockwise direction). The traditional DBSCAN algorithm continuously builds the polygon from a starting point and the result is a set of accidents. Consequently, there should be an additional step to give the corner points in the appropriate order. It is possible to do this after the DBSCAN finishes, but it is also possible to extend the DBSCAN method with the following steps:

- In the case of the first (*P*_1_) and second (*P*_2_) items, the concept of “polygon” cannot be interpreted. Hence, these are automatically marked as further corner points of the polygon.
- With the third point (*P*_3_), the items already form a polygon. The *p*_3_ point must be on the right side of the vector $\overleftarrow {{P_1}{P_2}}$, which can be checked using a scalar multiplication to ensure the clockwise direction requested by the Gauss formula. If this is not the case, it is necessary to change the order of *P*_1_ and *P*_2_. After that step, *P*_1_, *P*_2_ and *P*_3_ will be the corner points of the polygon in a clockwise direction.
- For every additional point (*P*_5_, *P*_6_, $\ldots$, *P*_*n*_), it must be checked that the additional point is inside the actual boundary convex polygon or not. It is possible to check that the new point (*P*_new_) is on the right side of the boundary vector or not. If it is true for each vectors, the point must be inside the polygon (or in the border). Therefore, it is not necessary to modify the shape. If the new point is on the left side of any boundary vectors, then it is outside the boundary convex polygon. There must be a sequence of one or more consecutive vectors breaking the rule. Let *k* and *l* be the first and last vectors of this sequence. It is possible to substitute the ${P_{k - 1}},{P_k},{P_{k + 1}}, \ldots ,{P_{l - 1}},{P_l},{P_{l + 1}}$ part of the boundary vector list with *P*_k__− 1_, *P*_new_, *P*_l__+ 1_. Because of the convexity of the original polygon, the *P*_k__− 1_, *P*_new_, *P*_l__+ 1_ triangle contains all the ${P_k},{P_{k + 1}}, \ldots ,{P_{l - 1}},{P_l}$ points, and the transformation also ensures the convexity of the new polygon and the clockwise direction of the corner points. Three figures about this process have been attached to the article in the Supplemental File “DBSCAN images”.

It is possible to calculate the black spot area and the accident density of a given cluster using the previous method.

### Analysis of black spot candidates

The result of the various black spot localization algorithms (sliding window, clustering, etc.) is a list of potential hot spots. However, having some accidents in a cluster does not mean that the hazard of accidents is significantly higher here. It is usually accepted by researchers that the number of accidents in a given area (section) of the road network fits the Poisson distribution. A special feature of road accident distribution is that the number of accidents is relatively low (compared to the size of the road network), and the variance is high. Therefore, the volatility of the accident number is very high, which means that a given cluster where the number of accidents is above the average is not inevitably a hot spot. The list given by the previous methods needs further examination to find the real hazardous sites.

At this point, the methodology of this paper significantly differs from the work of road safety engineers. Their objective is to find hazardous sites and take the appropriate actions to decrease the probability of further accidents. They must select the sites having the largest safety potential where the best cost-effective actions can be taken to decrease the number and severity of accidents. It is a very complex procedure based on the data of historical accidents, the expected number of accidents, the environmental conditions and the cost/expected benefits of different safety actions. Contrary to this, the objective of a self-driven car is not the elimination of road safety problems. As an ordinary participant of traffic, it has no chance to make the road network better. Nevertheless, as a passive participant, it should be able to localize the problematic areas, analyze these, and take the necessary preliminary steps to avoid further accidents.

Another difference between the methods of these fields is that from the perspective of road safety engineers, it is not necessary that the accidents of a given black spot have any special patterns or common characteristics. For the self-driven car, the localization of high-risk areas where the number of accidents is significantly higher than expected is not enough because this fact does not help to take the appropriate preliminary steps. This is the reason why this paper focuses on the identification of accident reasons.

The result of this further investigation can be one of the following:

- If it is not possible to identify any unexpected pattern in the accident attributes then the cluster cannot be considered as an accident black spot. The high number of accidents is just a coincidence and there are no suggestions to avoid further crashes.
- In contrast, if there is a special pattern in the accident attributes then this cluster has the potential to decrease the probability of further crashes. These reasons for similar accidents would be related to the road network, weather, lighting conditions or human errors (drivers and pedestrians).

In the second case, the knowledge of this special pattern (the common reasons for accidents in the same cluster) can be essential. It is presumable that it is possible to avoid accidents caused by the car itself. For example, if it is visible from the accident database that the number of accidents caused by slippery road is significantly higher than expected in a given area, the self-driven car should decrease the speed or change its trajectory to reduce the probability of this event. However, it is also worth noting that the preliminary actions can be very useful to decrease the probability of accidents caused by other drivers or pedestrians. For example, if the historical accident data contains patterns that the number of accidents caused by pedestrians is higher than expected, then the self-driven car would proactively try to decrease this negative potential using some type of visual or auditory warning or decreasing speed.

#### Deducing the environmental reasons for accidents

Accident databases usually contain certain taxonomy for accident types. These are usually structured classes of specific events and reasons, and scene investigators must classify each accident into one of these categories, which is very important statistical information. This method has several limitations because it is rare when the occurrence of an accident originates in one specific reason. Usually, multiple reasons, forming a complex structure, cause an accident. For example, the investigator codes the accident as a type of “catching-up accident”, but this does not give any information about why the accident occurs. It is also typical that most of the accidents in the Hungarian road network are caused by “incorrect choice of speed”. However, it is obvious that not just the speeding itself was the triggering reason for these accidents. There should be other factors (besides, it is unarguable that speeding increases the effects of other factors and makes certain accidents unavoidable).

Based on these experiences, this paper does not try to assign all accidents to mutually exclusive accident reason classes. Contrarily, the proposed method defines several potential accident reasons, which are not mutually exclusive. These factors can be complementary and having different weights and roles in the occurrence of the accident. Only the reasons with potential preventive operations are discussed because these have valuable information for the self-driven car.

The proposed method is based on the following consecutive steps:

1. All known accidents are analyzed by all possible accident reasons, and a score value is assigned to the accident showing how much the accident is affected by a given factor.
2. The distribution of these score values is approximated by the examination of all known accidents.
3. Based on the result of the previously presented DBSCAN algorithm, the distribution of these score values is also calculated for each black spot candidate.
4. The distributions for all accidents and a given black spot are compared. If the distribution of a given factor is significantly differing (to the positive direction), the cluster is marked as a hazardous area for the given factor.

The independent accident reason factors, like “slippery road”, “bad visibility”, “careless pedestrians”, etc. are defined as *R*_1_, *R*_2_,…, *R*_*N*_, where *N* is the number of these. As discussed previously, these reasons are not stored directly in the database but can be inferred from the general attributes of accidents. A scoring table is used for this purpose: the weights of the *i*-th accident factor (1 *≤ i ≤ N*) is stored as *W*^i^; where *W*^i^_attr__=__value_ shows the score for the *R*_i_ accident reason when the *attr* attribute equals to *value*.

Accordingly, the cumulative score of the *R*_i_ reason for the *x* accident is Eq. (3):

(3) $${S_i}(x) = \sum\limits_{\forall attr \in {\mathcal{A}}(x)} W_{attr = x.attr}^i$$

where *S*_i_(*x*) is the score value of the *R*_i_ reason for accident *x*. The *x.y* corresponds to the value of the specific *y* attribute of the *x* accident, and ${\mathcal{A}}(x)$ contains all the available known attributes of *x*.

It is also possible to calculate the same value, not just for an accident but also for all accidents of a black spot candidate. The *H*_i_(*C*) set contains all the *S*_i_(*x*) score values for all *x* accidents in the *C* cluster as visible in Eq. (4):

(4) $${H_i}(C) = \{ {S_i}(x)|x \in C\}$$

#### Distribution of accident scores

As a further step, it is necessary to determine that there is any significant reason which proves that the *C* set is a real hot spot or not. For a well-established decision, it is necessary to analyze all the accidents in the database to determine the main characteristics of the distributions of all *R* reasons. Based on these results, it is possible to compare the distributions of *H*_i_(*C*) values for the examined *C* hot spot candidate and the reference ${\hat H_i}$ values for the whole accident database (*D*) for a given *R*_i_ reason Eq. (5).

(5) $${\hat H_i} = \{ {S_i}(x)|x \in D\}$$

If the distributions of *H*_i_(*C*) and ${\hat H_i}$ are the same, it is assumable that the *R*_i_ reason has no significant role in the accumulation of accidents. Otherwise, if these distributions differ as the number *R*_i_ score values are higher in *H*_i_(*C*) than in ${\hat H_i}$, there may be some cause/causal relationship between them.

Hypothesis tests can show if the mean value of a given accident reason score (*R*_i_) in a given cluster is higher than the same mean for all accidents in the database. The used alternative hypothesis states that the mean score of the cluster minus the mean score of the whole population is greater than zero Eq. (7). The null hypothesis covers all other possible outcomes Eq. (6).

(6) $${_ \mathrel{\setbox0=\hbox{$\vdash$}
 \rlap{\raise.2ex\hbox to\wd0{\hss/\hss}}\box0} }:{\mu _C} - {\mu _D} \le 0$$

(7) $${_ \mathrel{\setbox0=\hbox{$\mathrel|\,\joinrel\mathrel|\joinrel\relbar$}
\rlap{\hbox to\wd0{\hss$\;$/\hss}}\box0} }:{\mu _C} - {\mu _D} > 0$$

where:

- μ_C_ is the mean score value for the black spot candidate;
- μ_D_ is the mean score value for all accidents in the database (full population).

This article proposes the application of Welch’s *t*-test, which is a two-sample location test used to test the hypothesis that the means of two populations are equal (like the popular Student’s *t*-test, but Welch’s test is more reliable when sample sizes are significantly different and the variances are also unequal). The Welch’s test assumes that both populations have normal distributions. Nevertheless, in the case of moderately large samples and application of the one-tailed test, the *t*-tests are relatively robust to moderate violations of the normality assumption. In this case, the populations are large enough (the full population contains thousands of accidents and black spots also contain several accidents), and it also holds that the one-tailed test is the appropriate method because we are looking for clusters where the mean is significantly higher than in the entire population. Ahad & Yahaya (2014) shows that Welch’s test can cause Type I errors when the variances of the two populations differ and distributions are non-normal. In this case, the variances are similar, and Type I errors are acceptable (some identified black spot candidates may not be real black spots).

According to Welch’s method, the statistic *t* value is given by Eq. (8).

(8) $$t = \displaystyle{{{{\overline x }_1} - {{\overline x }_2}} \over {\sqrt {\displaystyle{{{v_1}} \over {{n_1}}} + \displaystyle{{{v_2}} \over {{n_2}}}} }}$$

where:

- ${\overline x _1}$ is the mean of the first sample;
- ${\overline x _2}$ is the mean of the second sample;
- *v*_1_ is the variance of the first sample;
- *v*_2_ is the variance of the second sample;
- *n*_1_ is the size of the first sample;
- *n*_2_ is the size of the second sample.

The degree of freedom (*v*) is calculated by Eq. (9)

(9) $$v = \displaystyle{{{{\bigg(\displaystyle{{{v_1}} \over {{n_1}}} + \displaystyle{{{v_2}} \over {{n_2}}}\bigg)}^2}} \over {\displaystyle{{v_1^2} \over {n_1^2({n_1} - 1)}} + \displaystyle{{v_2^2} \over {n_2^2({n_2} - 1)}}}}$$

Based on the previously calculated *t* and *v* values, the *t*-distribution can be used to determine the probability (*P*). The one-tailed test is applied because it will answer the question that the mean of the cluster is significantly higher than the mean of the entire population. Based on *P* and a previously defined level of significance (α) it is possible to reject or not the null hypothesis.

In the case of rejection, it can be assumed that the examined accident reason is related to the accidents as one of the possible causal factors. If the null hypothesis cannot be rejected, there is no evidence for this.

### Scoring factors

The practical evaluation presented by this paper focuses on one specific accident reason (*N* = 1) the slippery road condition factor (*R*_1_).

The used accident database contains more than two hundred fields, in four categories:

- general accident attributes (date and time, location, nature, etc.);
- general environmental attributes (weather conditions, visibility, etc.);
- data about participants (was it a vehicle or pedestrian, speed, direction, etc.);
- data about injured persons (age of the injured person, etc.).

Weighting tables have been developed to estimate the effect of a given accident reason factor on the occurrence of the accident. It is possible to distinguish the following three type of accident properties, focusing on the slippery road condition accident factor:

- Some fields directly contain information about the examined factor. In this case, the “Road surface” property (abbreviated as *roadsrf*) of an accident has an option of “4-oily, slippery”. This is taken as the basis for further weights; the score value of this attribute is 1.0 (*W*^1^_roadsrf__= 4_ = 1.0), showing that the accident is highly affected by the slippery road condition factor. It is worth noting that it is not efficient making a binary decision about the examined factor based on this value because there are other values (“3-snowy”, “5-another staining”) having similar effects. This is reflected in the weight values.
- In some cases, there are no such direct fields, but it is possible to deduce information about a given factor from the already existing data. For example, in the case of the slippery road condition factor, the weather conditions (*wthr* property in the database) can help this process. In these cases, the score values assigned to different weather condition cases show an estimation of how much the given factor affected the occurrence of the accident. In the case of snowing (“6-snowy”), it would be higher (*W*^1^_wthr__= 4_ = 0.3) than for ideal conditions like “1-sunny” (*W*^1^_wthr__= 1_ = 0). It is also considered that in the case of accident nature “31-Slipping, carving, overturning on the road”, the slippery road factor influenced the results (*W*^1^_accnat__= 32_ = 0.2)
- The last group contains the fields without any relation to the examined factor. For example, fields like “Age of the driver” do not affect the results. The weights for all values of these fields are consequently zero.

The Supplemental File “Scoring tables” contains the given weight values for the affected fields. Weight values are based on a comprehensive literature review from the fields of road safety and road friction measurements (Wallman & Åström, 2001; Andersson et al., 2007; Sokolovskij, 2010; Colomb, Duthon & Laukkanen, 2017). However, some of the values are affected by the subjective experiments of the authors. It should take some further research to determine the most efficient weights.

## Results

### Accident database

This paper uses the official road accident database of Hungary, where data regarding accidents with personal injury are collected by the police. After some conversion and corrections, this dataset is handled by the Central Statistics Department. The completeness of the database is ensured by legislation, and participants of public road accidents with personal injury are obliged to report it to the police. A police officer starts the data collection on the spot by recording the most relevant data about the location and the main attributes of the accidents (participants, casualties, etc.). After 30 days, it is possible to refine the final injury level for all participants. After that finalization step, the Central Statistics Department collects and rechecks all records. Road safety engineers and researchers can use this database for their work.

The evaluation part of this paper is based on the accidents of this database from 1 January 2011 to 31 December 2018. It contains 128,767 accidents with personal injury classified into three categories: fatal, serious and slight. There are no accidents in the database without personal injury. Because of the high number of accidents and high computational demand of the clustering algorithm, this paper deals with two counties of Hungary: accidents of “Győr-Moson-Sopron” county was used to find the optimal parameters of the algorithm and “Heves” county was used as a control dataset.

### DBSCAN clustering

The input database for the clustering was the personal injury accidents of a given county of Hungary (“Győr-Moson-Sopron” county). This experiment was performed twice at two consecutive time intervals to measure the robustness of the method. The examined *t* interval contains the accidents which occurred in 1 January 2011–31 December 2014. and the $\hat t$ validation interval was 1 January 2015–1 December 2018. The number of accidents was 3,256 in the *t* interval (the *D* set contains these accidents) and 3,011 in the $\hat t$ interval (the $\hat D$ set contains these accidents).

In the hot spot search phase, the following DBSCAN parameters were used:

- ε value: 100 m;
- minimum accident count: five accidents;
- minimum accident density: 0.0001 accident/m^2^.

The result of this raw DBSCAN clustering method was 165 black spot candidates in the *t* interval and 152 black spot candidates in the $\hat t$ interval.

### Statistical test

Unlike traditional black spot searching methods, the next step is not the calculation of some safety potential index, but the determination of the different accident reason factors using the scoring method presented in Section. Considering the *R*_1_ slippery road condition factor, the *S*_1_(*x*) value is calculated for all *x* accidents. Most of these are not related to a slippery road surface reasons; so, *S*_1_ value for these is 0.

As a prerequisite for the Welch-test, a population of *S*_i_(*y*) values is generated where *y* stands for all accidents in the database. The main parameters of this sample are:

- number of items (*n*_1_): 3,256
- mean (${\overline x _1}$): 0.2438
- variance (*v*_1_): 0.1115

It is possible to calculate these values for all of them, iterating the overall black spot candidates. Based on the whole population comparison and the black spot candidates, the Welch-test was applied to get the statistical result values. According to the Welch-test, it is possible to use the Student distribution with these parameters and the given level of significance (α = 0.05) to reject the null hypothesis or not.

Table 1 shows the black spot candidates of the *t* interval where the null hypothesis was rejected because the mean of the *R*_1_ score for the given black spot candidate was significantly higher than the expected average. It can be assumed that these black spots are affected by the examined *R*_1_ factor.

**Table 1:** Accident black spots where the null hypothesis was rejected.

#	Location	Count	Mean	Variance	Prob.
1	LAT 47.6301/LON 16.7333	8	0.75	0.0857	0.000878
2	LAT 47.5956/LON 17.5872	11	0.55	0.0887	0.003629
3	LAT 47.3866/LON 17.8659	5	1.12	0.2820	0.010502
4	LAT 47.5708/LON 17.5790	6	0.56	0.1307	0.040157

**Figure 1:**
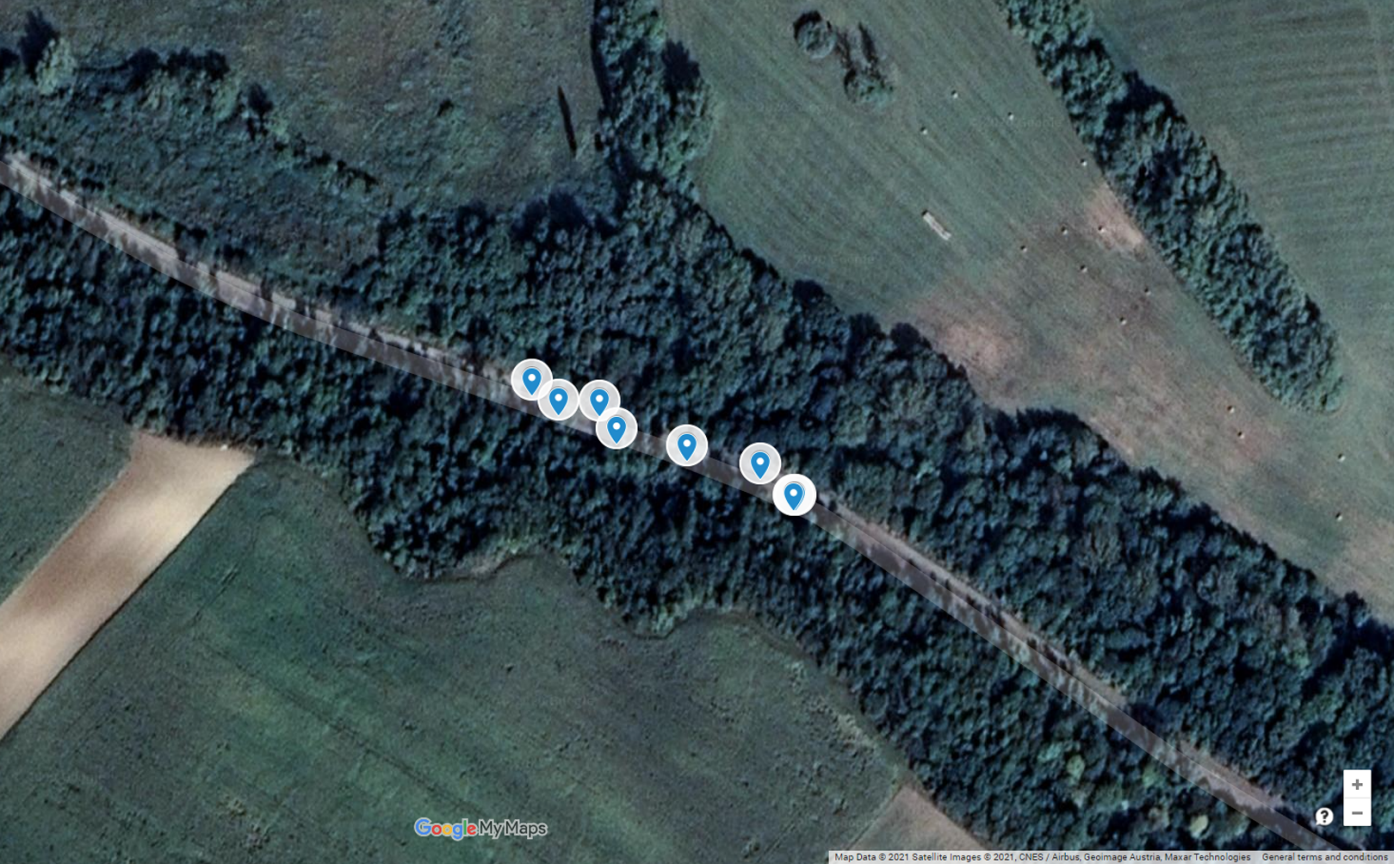
Road accidents of the black spot located at LAT 47.6301/LON 16.7333. Map Data @2021 Google, Satellite Images @2021 CNES/Airbus, Geoimage Austria, Maxar Technologies.

Figure 1 shows the environment and the accidents of the first black spot from this list. As is visible in the satellite image, it is a part of a long straight road; consequently, there is no reason for the autonomous car to decrease its speed. From the historical database, Table 2 contains detailed information about the accidents. As is visible, there is a high number of accidents affected by one or more slippery road-related attributes. This pattern significantly differs from the expectations; hence, there should be some environmental issues at this location. The examination and elimination of these reasons is the task of road safety experts (Orosz et al., 2015). Nevertheless, until then, it is worth taking preventive steps to decrease the chance of further accidents. The autonomous vehicle should adapt its control to this situation (speed reduction, using safer trajectory, etc.).

**Table 2:** Accidents of the black spot located at LAT 47.6301 / LON 16.7333.

Time	Latitude	Longitude	Outcome	Surface	Weather	Accident nature
2011.02.03 16:05	47.6302	16.7327	Light	Wet	Sunny	Track leaving
2011.05.06 17:35	47.6298	16.7340	Hard	Normal	Sunny	Track leaving
2011.06.26 10:24	47.6300	16.7338	Light	Wet	Rainy	Track leaving
2011.06.26 10:28	47.6300	16.7334	Hard	Wet	Rainy	Track leaving
2011.07.21 9:10	47.6302	16.7330	Hard	Wet	Rainy	Track leaving
2013.06.24 17:50	47.6298	16.7340	Light	Wet	Overcast	Frontal crash
2014.01.09 12:45	47.6301	16.7330	Light	Wet	Sunny	Slipping, carving
2014.01.20 10:45	47.6303	16.7325	Hard	Wet	Overcast	Track leaving

## Discussion

There is not any generally accepted method for the evaluation of black spots because there is not an exact definition for these. Based on real-world accident data, there is not any list of real black spots. So, the widely accepted confusion table-based methods are not usable here (assigning the clusters into true-positive, false-positive, true-negative, false-negative classes and calculating the common measurements like accuracy, recall, etc.). Therefore, it is necessary to evaluate these results based on the general characteristics of these locations. The accident density of black spots is significantly higher than the average; though, this is just a necessary condition but not sufficient for validity. Because of the high volatility of accidents, the regression to the mean effect can distort the results. It is a well-known statistical phenomenon that roads with a high number of road accidents in a particular period are likely to have fewer during the consecutive period just because of the random fluctuations in crash numbers. In the case of real black spots, the high number of accidents is permanent. Thus, it should be a good evaluation technique to check the number of accidents of the consecutive validation time interval inside the clusters identified in the *t* interval.

There are specific tests for this purpose introduced by Cheng & Washington (2005) used by various article (Montella, 2010): site consistency tests, method consistency tests, and the total rank differences test. Since these are developed for black spot searching methods based on road intervals, it was necessary to adapt them to use spatial coordinates and black spot regions. The input series for all tests were the result of the previous black spot identification process, as

- *C*_i_ is the *i*-th cluster identified in the *D* database (1 *≤ i ≤ n* where *n* is the number of identified black spots in the *t* interval);
- ${\hat C_i}$ is the *i*-th cluster identified in the $\hat D$ database ($1 \le i \le \hat n$ where $\hat n$ is the number of identified black spots in the $\hat t$ interval).

### Site consistency test

This test assumes that any site identified as a black spot in the *t* time period should also reveal high risk in the subsequent $\hat t$ time period.

Let π(*C*) the convex boundary polygon of the *C* cluster given by the algorithm presented in Section, and π is the union of these regions identified in the *t* time period (10).

(10) $$\Pi = \bigcup\limits_{i = 1}^n \Pi ({C_i})$$

As the next step, we collect all accidents for the consecutive $\hat t$ time period, which are inside the clusters identified by the prior *t* time period. The *T*_1_ attribute shows the number of these accidents divided by the summarized area of these clusters. Thus, this is the accident density of these clusters in the consecutive time period Eq. (11).

(11) $${T_1} = \displaystyle{{|\{ x \in \hat D|x\;inside\;\Pi \} |} \over {\sum\limits_{i = 1}^n {\rm \alpha} ({C_i})}}$$

### Accident reason factor consistency test

As this paper goes further, revealing the accident reason factors, it is also worth checking if the accidents in the $\hat t$ time period inside the region identified by the *t* time period have the same attributes or not. This leads to the introduction of the *T*′_1_ value, which shows the average score value for these accidents Eq. (12).

(12) $${{T}^{\prime}_1} = \displaystyle{{\sum\nolimits_{\forall x \in \hat D} \bigg\{ {\matrix{ {{S_1}(x),} & {if\;x\;inside\;\Pi } \cr {0,} & {else} \cr } } } \over {|\{ x \in \hat D|x\;inside\;\Pi \} |}}$$

### Method consistency test

It is also assumable that a black spot area identified in the *t* time period will also be identified as black spot in the consecutive $\hat t$ time period. A given black spot searching method can be considered consistent if the number of a black spots identified in both periods is large. Meanwhile, that of black spots identified only in one of the examined periods is small. It is possible to use Eq. (13) to calculate this method consistency:

(13) $${T_2} = \displaystyle{{|\{ {C_1},{C_2}, \ldots {C_n}\} \cap \{ {{\hat C}_1},{{\hat C}_2}, \ldots {{\hat C}_{\hat n}}\} |} \over {|\{ {C_1},{C_2}, \ldots {C_n}\} \triangle\{ {{\hat C}_1},{{\hat C}_2}, \ldots {{\hat C}_{\hat n}}\} |}}$$

where *T*_2_ is the ratio of the number of clusters existing in both search results and the number of clusters given by only the search in *t* or only in $\hat t$ time period (△ stands for the symmetric difference of sets). A pair of clusters from the *t* and $\hat t$ period considered identical if the distance between these is less than 300 m.

### Rank difference test

The rank difference test is based on black spots identified in both the *t* and $\hat t$ periods. The black spots of both periods are sorted by accident density, and the rank difference test shows the difference in the positions of the same cluster in the two lists. The smaller the value, the more consistent the examined method is, because the sequence of clusters is similar. Large numbers shows that the examined method was able to identify the same black spot in both intervals but with a different severity related to each other.

Let *O* and $\hat O$ the sequences of black spots identified in both periods (both sequences contain the items of the $\{ {C_1},{C_2}, \ldots {C_n}\} \cap \{ {\hat C_1},{\hat C_2}, \ldots {\hat C_{\hat n}}\}$ set) ordered by accident density in the *t* time period (*O*) and in the $\hat t$ time period ($\hat O$). The *T*_3_ will show the rank difference of the examined method Eq. (14). Obviously $|O| = |\hat O|$.

(14) $${T_3} = \displaystyle{{\sum\nolimits_{c \in O} |Rank(c,O) - Rank(c,\hat O)|} \over {|O|}}$$

where *Rank*(*x*, *Y*) is the rank of the *x* black spot in the *Y* sequence.

### Evaluation results

First, the proposed method was compared to the traditional Sliding Window method (SW) using dynamic window length. The minimal window length parameter was 250 m, the minimal accident number was 5, and the minimal accident density was 0.01 accidents/m. As a further step, the novel method was also compared to the raw DBSCAN based clustering (without the accident factor scoring). The parameters of this were the same as presented above. The proposed method is presented in the comparison under the DARF (DBSCAN with Accident Reason Factor determination) name.

Table 3 shows the overall results for “Győr-Moson-Sopron” county. As visible, the number of black spots recognized by the DARF method is significantly less than by its alternatives. It was expected because the SW and DBSCAN methods list all clusters where the accident density is higher than a given threshold. In contrast, the DARF method results in only black spots affected by the *R*_1_ accident factor. The difference between the SW and DBSCAN is also significant and is caused by the fact that the SW uses road name + road section positioning which is not available in built-up areas. In comparison, the DBSCAN method is based on GPS coordinates and can find the black spots of municipal roads (which is one advantage of this approach).

**Table 3:** Results of the comparison of the SW, DBSCAN, and DARF methods based on the road slippery condition. Precision is the ratio of the number of confirmed black spots (identified in both intervals) and the number of all black spots (identified at least in one of the intervals). Results are based on the personal injury accidents occured in “Győr-Moson-Sopron” county.

Value	SW	DBSCAN	DARF
BS identified in both *t* and $\hat{t}$	67	129	4
BS identified in *t* but not in $\hat{t}$	8	36	2
BS identified in $\hat{t}$ but not in *t*	20	23	0
Precision	41.36%	40.69%	40.00%
*T*_1_ test result (accidents/m)	0.0094	0.0435	0.0447
*T*′_1_ test result	0.2159	0.1922	0.6200
*T*_2_ test result	0.5447	0.5223	0.5000
*T*_3_ test result	3.8765	5.9054	0.2000

The *T*_1_ result is similar in the case of DBSCAN and DARF methods and it is significantly less in the case of SW. The *T*_2_ results are almost the same for all algorithms. The third general metric shows that the proposed method performs very well on the rank difference test. However, it is worth noting that the number of black spots is significantly less in this case, which can be an advantage.

The *T*′_1_ metric shows the real strength of the proposed method. As expected, black spots identified by the SW and DBSCAN contain a mixture of various accidents. Consequently, the average of the *R*_1_ score is near to the mean of the population (0.2159 and 0.1922 compared to 0.2438). Contrary to this, the score number for the accidents of the $\hat t$ time interval placed inside the clusters located by the data of the *t* interval is 0.62, which is significantly higher than the average.

These results confirm that the proposed method has very similar characteristics to the already existing methods. The slightly lower *T*_2_ value shows that as a raw black spot searching algorithm, it is not as robust as the alternatives. Nonetheless, the *T*′_1_ result shows that it is satisfactory for our purpose. It can localize areas when the expected number of accidents with given accident reasons is significantly higher than the average.

Table 4 shows the same values for another county (“Heves”) as a control dataset to check the robustness of the method. As visible, the main characteristics of the results are very similar. In this case, the *T*_1_ and *T*_3_ results are better compared to the alternatives. However, the *T*′_1_ value is slightly lower, but still significantly higher than the population average.

**Table 4:** Results of the comparison of the SW, DBSCAN, and DARF methods based on the road slippery condition. Precision is the ratio of the number of confirmed black spots (identified in both intervals) and the number of all black spots (identified at least in one of the intervals). Results are based on the personal injury accidents are occured in “Heves” county.

Value	SW	DBSCAN	DARF
BS identified in both *t* and $\hat{t}$	25	38	4
BS identified in *t* but not in $\hat{t}$	9	12	0
BS identified in $\hat{t}$ but not in *t*	16	26	3
Precision	33.33%	33.33%	36.36%
*T*_1_ test result (accidents/m)	0.0074	0.0323	0.0732
*T*′_1_ test result	0.2148	0.2286	0.5778
*T*_2_ test result	0.3333	0.3333	0.4000
*T*_3_ test result	1.8667	1.4912	0.0000

## Conclusions

This work presents a novel, fully automated method updating autonomous vehicles concerning potential road risk factors. The method is based on the DBSCAN data-mining algorithm, which can localize black spot candidates where the number of accidents is greater than expected. It has several advantages to the traditional sliding window method, especially in built-up areas and accidents occurred at junctions.

Beyond the traditional road safety engineering work, an additional processing step was also introduced, making assumptions about the main accident reasons. All possible reasons (road slippery, pedestrian issues, etc.) should be checked one-by-one, assigning score values to all accidents. The proposed method considers the distribution of these score values for the full population (all accidents of the given county) and each black spot candidate. Using hypotheses tests (one-tailed Welch-test), it is possible to select clusters in which the mean of the score values is significantly higher than the expected value (calculated by statistical methods based on the entire accident database). These can be considered as black spots affected by the given factor.

The output of this process is a sequence of risky locations on the public road network and a prediction concerning the accident reasons. These would be the base of further research suggesting automatic preventive steps to autonomous vehicles. This dataset can be useful in the route planning phase (try to avoid black spots) and in the traveling phase (take preventive steps when approaching dangerous locations) (Alonso et al., 2016). This knowledge would decrease the number and seriousness of public road accidents.

As a limitation, it is worth noting that the proposed method would result in false positive alarms. Fortunately, these results are used by autonomous vehicles; therefore, the consequences are usually minor inconveniences (decreasing the speed, etc.) compared to the traditional road safety investigations, where the manual revision is essential. It is also worth seeing that our method is based only on local historical data resulting in problems typical of traditional statistical black spot searching methods (high variation compared to the expected value). It would be worth developing a hybrid method based on the Empirical Bayes method, which achieves superior control for random variation.

The next step of this research project will be the development of these preventive steps. The previously acquired information should be built into the control of the self-driven vehicle to fine-tune its strategy of movement to avoid all predictable risky situations. For example, if the presented method predicts high probability of pedestrian accidents, the car should increase the engine voice volume; in the case of a high chance of frontal accidents, it is worth increasing the power of the headlights; and obviously, decreasing the speed near any of the dangerous locations may decrease the seriousness of most accidents. Building an expert system to give similar advice based on the historical data should be the next step of this project.

Another direction of further development is to make the method more sensitive to real-time environmental conditions. For example, if the autonomous car has to plan a route at night in wet weather, then it should pay more attention to historical accidents that have occurred under similar conditions. This also confirms the fact that it is necessary to make simple and fully automatic algorithms for this purpose to make the fast recalculations available.

As another further development, an Artificial Intelligence based approach should be used to extend the database to solve the problems raised by the limitations of the dataset.

## Supplemental Information

10.7717/peerj-cs.399/supp-1 Supplemental Information 1 Accident database. Each row of the file stands for an accident.

10.7717/peerj-cs.399/supp-2 Supplemental Information 2 Accident scoring. Contains multiple tables with headers.

10.7717/peerj-cs.399/supp-3 Supplemental Information 3 Code.

10.7717/peerj-cs.399/supp-4 Supplemental Information 4 DBSCAN Images.

## Additional Information and Declarations

### Data Availability

The following information was supplied regarding data availability: Data and code are available in the Supplemental Files.
