# Supplementary material for: Analysis of historical road accident data supporting autonomous vehicle control strategies
Source: PeerJ Comput Sci. 2021 Feb 23;7:e399. doi: 10.7717/peerj-cs.399 (PMC7959616; doi:10.7717/peerj-cs.399)
Supplement: Supplemental Information 2 — Contains multiple tables with headers. [file peerj-cs-07-399-s002.pdf]

Table 1: "Slippery Road Surface" (R_1) scores for the "Accident Nature" property

Code	Description	Weight
31	Slipping, carving, overturning on the road	$W^1_{accnat=31} = 0.9$
32	Track leaving, with solid object collision	$W^1_{accnat=32} = 0.4$
33	Track leaving, without solid object collision	$WS^1_{accnat=33} = 0.4$
?	Other cases	$W^1_{accnat=?} = 0.0$

Table 2: "Slippery Road Surface" (R_1) scores for the "Road Surface" property

Code	Description	Weight
1	normal	$W^1_{roadsrf=1} = 0.0$
2	wet	$W^1_{roadsrf=2} = 0.3$
3	snowy	$W^1_{roadsrf=3} = 0.6$
4	oily, slippery	$W^1_{roadsrf=4} = 1.0$
5	other staining (mud, etc.)	$W^1_{roadsrf=5} = 0.7$
6	not a road	$W^1_{roadsrf=6} = 0.0$

Table 3: "Slippery Road Surface" (R_1) scores for the "Weather" property

Code	Description	Weight
1	sunny	$W^1_{wthr=1} = 0.0$
2	overcast	$W^1_{wthr=2} = 0.0$
3	foggy	$W^1_{wthr=3} = 0.1$
4	rainy	$W^1_{wthr=4} = 0.2$
5	stormy	$W^1_{wthr=5} = 0.3$
6	snowy	$W^1_{wthr=6} = 0.5$
