# Supplementary material for: Analysis of historical road accident data supporting autonomous vehicle control strategies
Source: PeerJ Comput Sci. 2021 Feb 23;7:e399. doi: 10.7717/peerj-cs.399 (PMC7959616; doi:10.7717/peerj-cs.399)
Supplement: Supplemental Information 4 [file peerj-cs-07-399-s004.pdf]

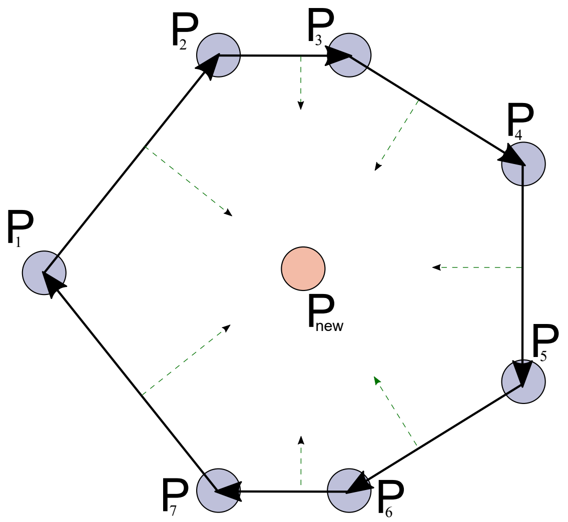

(a) The new point is inside the boundary convex polygon.
It is on the right side of all vectors (green arrows).

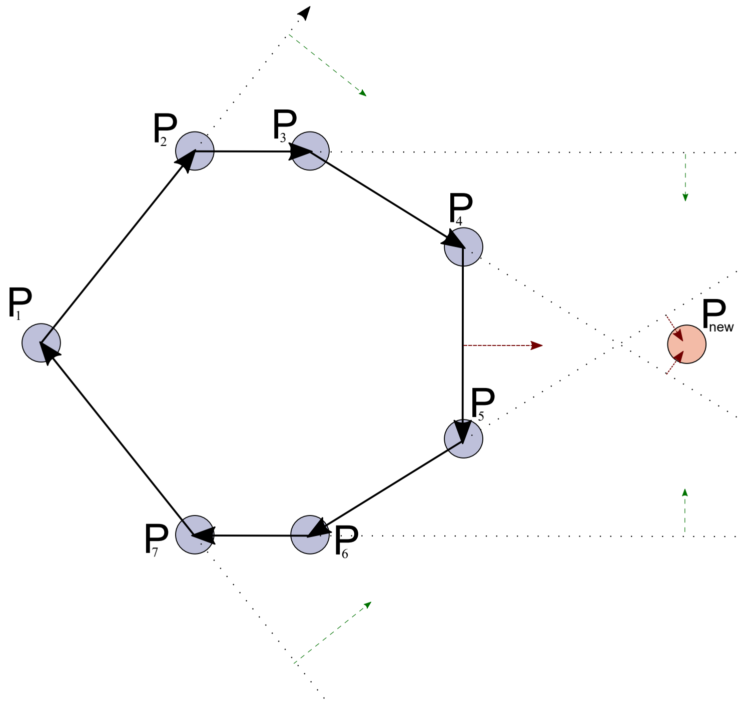

(b) The new point is outside the boundary convex polygon. In some cases,
it is on the right side of the boundary vectors (red arrows).

Figure 1: Checking whether the new point is inside or outside the already existing polygon.

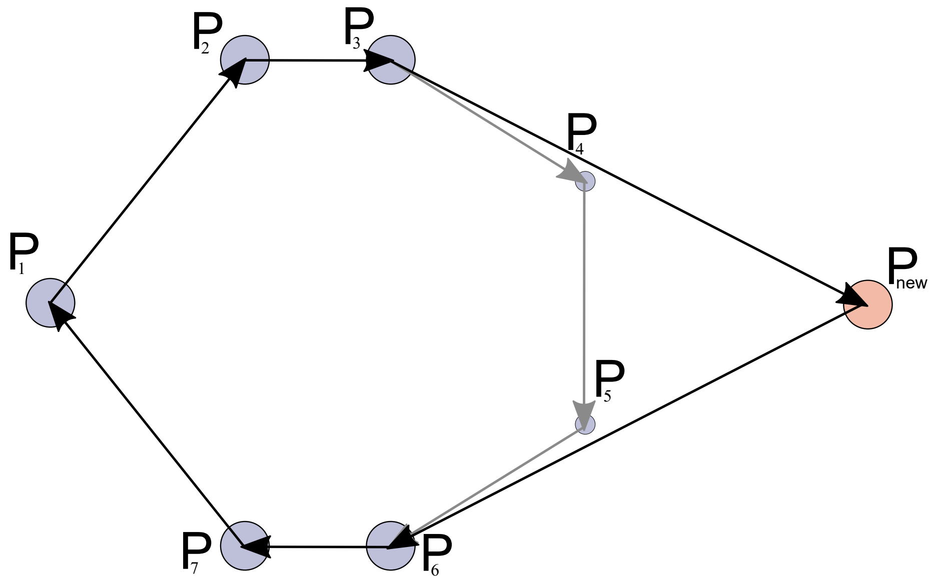

Figure 2: Modifying the polygon to contain the new point. The P_4 and P_5 points are no more the part of the border, these becomes inner points. The P_{new} point becomes a new point of the border, extending the area of the cluster.
